# Microbiota–host communications: Bacterial extracellular vesicles as a common language

**DOI:** 10.1371/journal.ppat.1009508

**Published:** 2021-05-13

**Authors:** Rogers A. Ñahui Palomino, Christophe Vanpouille, Paolo E. Costantini, Leonid Margolis

**Affiliations:** 1 Section on Intercellular Interaction, Eunice Kennedy Shriver National Institute of Child Health and Human Development, National Institutes of Health, Bethesda, Maryland, United States of America; 2 Department of Pharmacy and Biotechnology, University of Bologna, Bologna, Italy; UNITED STATES

## Abstract

Both gram-negative and gram-positive bacteria release extracellular vesicles (EVs) that contain components from their mother cells. Bacterial EVs are similar in size to mammalian-derived EVs and are thought to mediate bacteria–host communications by transporting diverse bioactive molecules including proteins, nucleic acids, lipids, and metabolites. Bacterial EVs have been implicated in bacteria–bacteria and bacteria–host interactions, promoting health or causing various pathologies. Although the science of bacterial EVs is less developed than that of eukaryotic EVs, the number of studies on bacterial EVs is continuously increasing. This review highlights the current state of knowledge in the rapidly evolving field of bacterial EV science, focusing on their discovery, isolation, biogenesis, and more specifically on their role in microbiota–host communications. Knowledge of these mechanisms may be translated into new therapeutics and diagnostics based on bacterial EVs.

## Introduction

During and after a mammal’s birth, bacteria move into its body, forming part of the microbiota. To communicate and probably to survive, bacteria and mammalian cells have to establish a common language, just as groups of humans have to learn each other’s language when moving to the same territory. An essential part of this common language of mammalian cell–cell communication is thought to be through extracellular vesicles (EVs). Both bacteria and mammalian cells release EVs. Remarkably, in spite of the huge differences between bacterial and mammalian cells in size, structure, metabolism, and general physiology, the EVs they release are essentially of the same size.

EVs are nano-sized vesicles released by life forms of all domains under both normal and pathological conditions (reviewed in [1–4]). The roles of EVs, and in particular bacterial EVs, in promoting health and in causing various pathologies, whether through bacterial–bacterial or bacterial–host interactions, are becoming increasingly evident.

EVs are widely studied in vitro in laboratories, using cell lines. In contrast to EVs isolated from cell line cultures, in vivo EVs isolated from various human body fluids are mixtures of vesicles released by cells of different types. Since bacteria also generate EVs, one cannot exclude the possibility that a fraction of body fluid–derived EVs also contains EVs generated by bacteria that normally colonize human mucosae. In fact, the majority, if not all, of the studies done on EVs derived from human body fluids do not take in consideration these bacterial EVs [5], although they most likely interact with host cells. Thus, to study EVs derived from body fluids, one has first to separate the bacterial EVs from the mammalian ones. However, such separation constitutes a significant challenge, as bacterial EVs range between 20 and 400 nm in diameter, similar in size to EVs derived from eukaryotic cells [6–12]. Therefore, these 2 types of EVs cannot be separated on the basis of size differences. Moreover, the difficulty of distinguishing bacterial EVs from eukaryotic EVs also stems from our lack of knowledge of specific bacterial EV markers and from lack of a deep understanding of the cargo of bacterial EVs as well as of their biogenesis. As the result, the pattern and mechanisms of interactions between bacterial EVs and host cells are mostly unknown.

Both gram-negative and gram-positive bacteria secrete vesicles that contain components from their mother cells, as has also been observed for EVs in other domains of life. Like mammalian EVs, bacterial EVs are thought to mediate cell–cell communications by transporting diverse bioactive molecules including proteins, nucleic acids, lipids, and metabolites. In this review, we discuss how bacteria produce EVs and how these EVs mediate communications between bacteria and mammalian host.

## Bacterial EV discovery

As with the history of mammalian EVs, occasional reports on bacterial EVs can be traced in the scientific literature back to the 1960s (see [13]). It is difficult to trace the discovery of bacterial EVs to a particular researcher, since bacterial EVs are easily observed in regular electron microscopy and were seen by many observers. The problem was not in discovering EVs but rather in understanding what the biological meaning and functions of these EVs are. In particular, since polar lipids in aqueous solution tend to form vesicular structures, it was thought that EVs constitute cellular debris from decomposition of dead cells, in particular of their lipid membranes. However, the fact that EV production by bacteria requires their metabolic activity [8,10,14] together with data showing that bacterial EVs share many similarities in structure and function with mammalian EVs constitute strong evidence that EVs are released by living bacteria.

On the basis of morphology, structural organization, and differential staining properties, bacteria are classed as gram-positive or gram-negative. A gram-positive bacterium has a thick cell wall rich in peptidoglycans, while a gram-negative bacterium has 2 membranes, an outer and an inner one. The outer membrane contains lipopolysaccharides (LPS). The majority of our knowledge of bacterial EVs has been obtained from studies of gram-negative bacteria, whereas EVs released by gram-positive bacteria constitute a relatively new field of research, as it has always been difficult to imagine how EVs could be released through the gram-positive bacterium’s thick wall. Even when in mid-1990s, vesicle-like blebs were described on the surface of gram-positive *Bacillus* spp., their nature was not investigated further [15].

**Fig 1:**
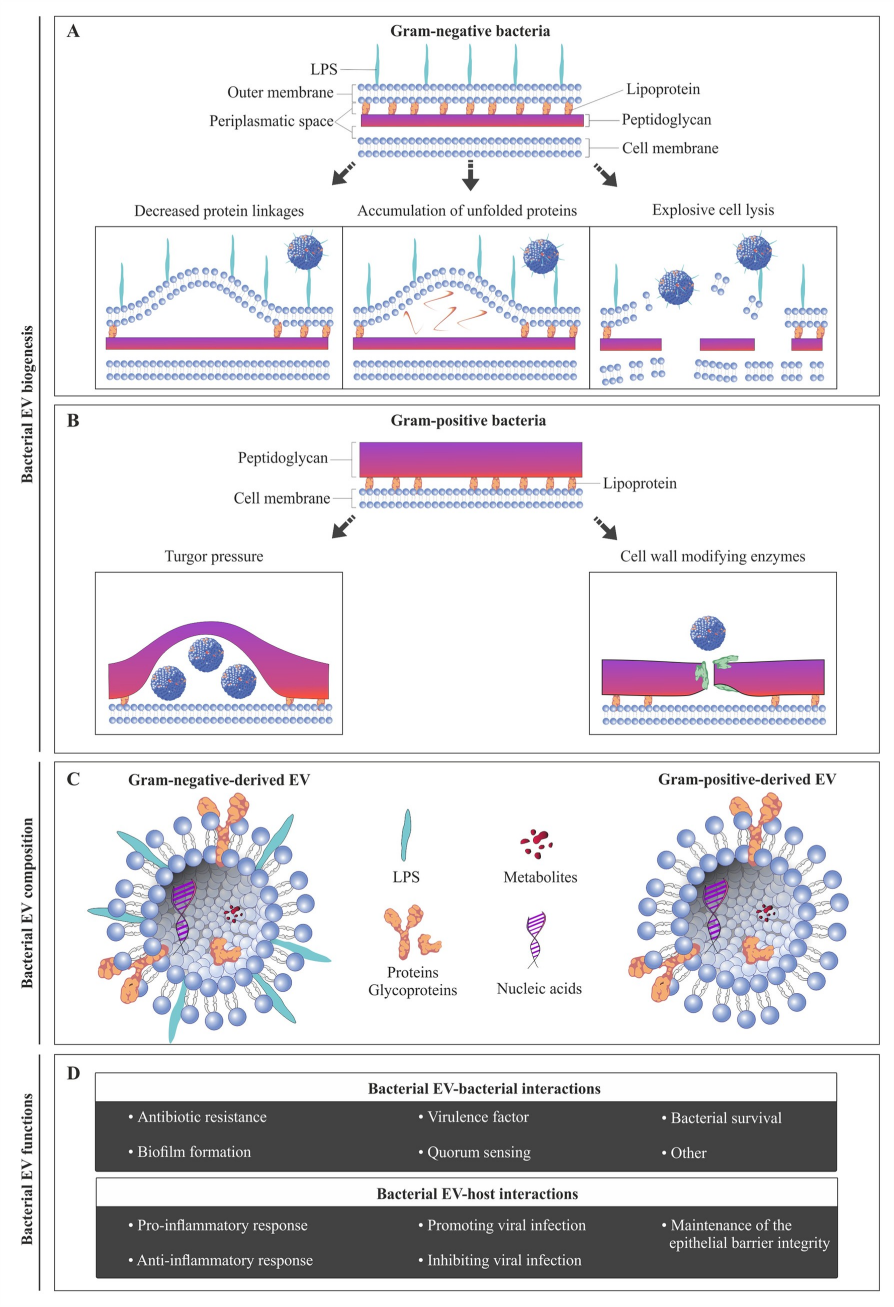
Bacterial EV biogenesis, composition, and functions. **(A)** EVs derived from gram-negative bacteria can be released through the outer membrane (i) by decreased protein linkages between the outer membrane and peptidoglycan; (ii) by accumulation of unfolded proteins and/or fragments of peptidoglycan in the periplasmic space generating turgor pressure; and (iii) by explosive cell lysis. **(B)** EVs derived from gram-positive bacteria can be released through the cell wall (i) by turgor pressure caused by the accumulation of EVs; and (ii) by the action of cell wall–degrading enzymes. **(C)** Bacterial EV composition includes a double phospholipidic layer, proteins, glycoproteins, metabolites, and nucleic acids. Gram-negative EVs differentiate from gram-positive-derived EVs by the presence of LPS on their surface. **(D)** EV functions during the interactions between bacteria or host cells. EVs, extracellular vesicles; LPS, lipopolysaccharides.

Remnants of this biased trend in the scientific literature continue, and while the literature on EVs from gram-negative bacteria, often called outer-membrane vesicles (OMVs), is rapidly growing, there are significantly fewer publications on EVs from gram-positive bacteria. Nevertheless, the biogenesis of vesicles derived from gram-positive and from gram-negative bacteria is likely different (Fig 1A and 1B). Gram-positive and gram-negative bacterial EVs are also different in their composition (Fig 1C). For example, since gram-positive bacteria do not contain LPS, EVs derived from these bacteria do not contain them either [13] (Fig 1C). The difference in composition of these EVs goes beyond the presence of LPS and includes other lipids, proteins, and EV cargo that ultimately result in the differences in functions of these EVs. While below we report on our knowledge on EVs from both types of bacteria separately, for consistency we will refer to both of them as bacterial EVs.

## EV isolation

Bacterial EVs have been isolated from various in vitro and in vivo sources [10,16,17]. Generally, cells are first pelleted by centrifugation, and the supernatants are filtered with 0.22-μm filters to eliminate from the samples any remaining bacteria. Then, bacterial EVs are isolated by ultracentrifugation, leading to a heterogeneous population of EVs that could be further fractionated by sucrose or iodixanol gradient centrifugation [18].

Bacterial EVs are negatively charged and contain Mg2+ or Ca2+ cations, which stabilize the surface charges and are relatively stable after purification. Bacterial EVs, like mammalian-derived EVs, seem to maintain their physicochemical properties in long-term storage at 4°C or at −80°C, even under multiple freeze–thaw cycles [19,20]. However, whether the EV surface composition is fully preserved under various storage conditions has not been studied.

## Bacterial EV composition, structure, and function

Considerable efforts have been undertaken to decipher the structure and to identify functions of EVs generated by commensal or pathogenic bacteria and to study their interactions with host cells [5,9,10]. As mentioned above, most studies on bacterial EVs were done with pathogenic bacteria from the gram-negative group.

In general, bacterial EVs are composed of a bilayer lipid membrane in which various proteins and glycoproteins are incorporated. Also, bacterial EVs contain various proteins, including enzymes and toxins, as well as nucleic acids inside the vesicles. Such a general description does not permit a distinction between mammalian and bacterial EVs.

While the composition and structure of EVs continue to be investigated by use of various techniques, including electron microscopy, mass spectroscopy, proteomic analysis, etc., deciphering various possible functions of bacterial EVs is a challenge at a higher level. A fruitful approach to investigate the role of EVs is probably to study the bioactivity of bacterial cargo associated with these EVs, whether they derive from gram-positive or gram-negative bacteria. Below, we discuss this approach in relation to gram-positive and to gram-negative bacteria separately.

### EVs derived from gram-negative bacteria

The release of EVs from gram-negative bacteria such as *Bacteroides* and *Escherichia coli* was evidenced during the 1960s from electron microscopy [21,22]. Although the number of in vivo and in vitro studies of EVs derived from gram-negative bacteria is rapidly increasing (reviewed in [9,23,24]), the mechanisms regulating EV release in these bacteria still remain hypothetical. The differences in bacterial structure and physiology dictate different pathways of EV release, which in turn may lead to distinct types of EVs (reviewed in [24]).

In particular, gram-negative bacteria are characterized by a double plasma membrane layer separated by the periplasm. Most gram-negative–derived EVs, often called OMVs, bleb from the outer membrane and contain periplasmic contents including outer membrane proteins, lipoproteins, and lipids (reviewed in [11]). Some pathogenic gram-negative bacteria such as *Shewanella vesicular* M7^T^ also produce EVs of a different type, called inner membrane vesicles (IMVs). These vesicles are formed by fission of a protrusion of the outer and plasma membranes and thus entrap cytoplasm components including DNA and ATP [25,26]. Also, it has been found that the release of EVs increases under general stress responses [27].

Various models of EV biogenesis from gram-negative bacteria have been suggested by several authors [12,28–37] (Fig 1): (i) EVs are released when the outer membrane lipid asymmetry is compromised, causing membrane curvatures, thus facilitating its vesiculation. This asymmetry may originate from a decrease in protein linkages between the outer membrane and peptidoglycan or the shape of the transmembrane proteins; (ii) EVs are released by turgor pressure produced by the accumulations of unfolded proteins and/or fragments of peptidoglycans in the periplasmic space; (iii) the stability of the outer membrane is critically determined by interactions between LPS molecules on the surface of the outer membrane, with the number and strength of these LPS–LPS interactions relying on the structure of LPS itself and on salt bridges formed by cations [28]; and (iv) EVs are released as the result of explosive cell lysis [38]. Future studies will reveal by which of these mechanisms EVs are predominantly released from gram-negative bacteria.

### EVs derived from gram-positive bacteria

For many years, the consensus was that EVs could not be released through the thick cell wall of gram-positive bacteria. Their existence, however, was reported for the first time in 1990 in electron microscopy studies performed on *Bacillus cereus* and *Bacillus subtilis* [15]. Thereafter, the majority of studies reporting on EVs derived from gram-positive bacteria focused on *Staphylococcus aureus* [6] and bacteria of the phyla Firmicutes and Actinobacteria [10].

Despite an increasing number of publications, the mechanisms underlying the biogenesis of gram-positive EVs remain to be clarified. The current hypotheses explaining the release of EVs through the cell wall are (Fig 1) the following: (i) Turgor pressure on the cell wall caused by the accumulation of EVs, resulting in their release by the plasma membrane; (ii) degradation of the cell wall by the presence of cell wall–modifying enzymes; (iii) it is possible, although never proven, that the deformation of EVs allows their passage through pores that are narrower than the measured EV diameter (reviewed in [13,39]).

## Bacterial EVs and interaction between bacteria

Inside the microbial community, bacterial cells establish complex interactions with each other and EVs play an important role in both cooperation and competition strategies [40] (Fig 1D). Indeed, the diversity in cargo of bacterial EVs points out their roles in antibiotic resistance [7], biofilm formation [41,42], survival [43], virulence factor [36,44], quorum sensing [45,46], and other bacteria–bacteria communications [35]. In general, most of the specific bacterial EV cargo components responsible of a particular EV function have not been fully elucidated. We report in Table 1 some of the EV cargo component with proven function in in vivo or in vitro studies [6,7,9,36,41,42,45–53].

**Table 1:** Bacterial EV cargo components and their functions.

Function	Bacterial EV cargo
Antibiotic resistance	β-lactamase [47,48], BlaZ [7], and cephalosporinases [55] Transferring carbapenemase gene (OXA-24 gene) [49] Polymyxin B, colistin, and ampicillin EV entrapment [6,50]
Biofilm formation	Alkaline protease, PrpL, [41], and CdrA [42]
Survival	Antimicrobial quinolines [45] and hemin-binding protein C [9]
Virulence factor	Toxins and degradative enzymes (phospholipase C, alkaline phosphatase, serine protease, esterase lipase, cholera toxin, adenylate cyclase toxin, and VacA) [9,36]
Quorum sensing	PQS [45] and N-hexadecanoyl-L-homoserine lactone [46]
Decoy against bacteriophages	By binding LPS present in EVs [50] By neutralizing phages [51]
Killing competing bacteria	Endopeptidase L5, murein hydrolase, and peptidoglycan hydrolase [9]
Bacteria adhesion and invasion	Adhesin/invasion and OmpA [9]
Host immunomodulation	Cytolysin A, α-Hemolysin, VacA toxin, CNF1, enterotoxin, Shiga toxin LPS, PspA, and peptidoglycan [9,52,53]

EV, extracellular vesicle; LPS, lipopolysaccharides.

Antibiotic resistance is a fundamental characteristic of microbial communities, allowing them to continue populating the human body during drug treatment. Several studies have reported that bacterial EVs mediate this resistance through different mechanisms, such as horizontal gene transfer [54], EV entrapment of extra- and intracellular antimicrobials, and presence of enzymes for antibiotic resistance associated with EVs. This latter mechanism is mediated by EVs derived from both gram-positive and gram-negative bacteria. It allows the survival of the producer strain and the protection of other bacteria that may not be equipped with resistance enzyme. For example, *S*. *aureus*–derived EVs carrying β-lactamase have been shown to confer ampicillin resistance to *Salmonella enterica*, *E*. *coli*, and *Staphylococcus epidermis* [7]. Similarly, in gram-negative bacteria, *Haemophilus influenzae*–derived EVs containing β-lactamase protect group A streptococci from amoxicillin [47]. Species of *Bacteroides*, a predominant genus in the gut microbial community, protect pathogens and commensal bacteria against antibiotics of the β-lactam type by secreting EVs containing cephalosporinases [55]. Moreover, the antibiotic resistance enzymes associated with EVs are protected from possible inactivation [20]. In *Moraxella catarrhalis*, for example, the packaging of β-lactamase inside the EVs protects the enzyme from IgG, which usually decreases bacterial antibiotic resistance through β-lactamase neutralization [48].

Antibiotic resistance can also be pursued via horizontal gene transfer, transferring antibiotic resistance genes in EVs [10]. *Acinetobacter baumannii*, a carbapenem-resistant strain, is able to transfer the gene of *carbapenemase* in EVs to other *A*. *baumanii* strains not resistant to carbapenem, thus conferring on them resistance to carbapenems [49]. The use of EVs in horizontal gene transfer is not only a delivery system but also a way to prevent DNA thermo-degradation and degradation by nucleases [56,57].

Bacterial EVs may protect bacteria from various antibiotics as they also bind/capture antibiotics in the extracellular compartment, thus protecting the microbial community. This was demonstrated through use of a hyper-vesiculating mutant *E*. *coli* that releases more EVs than the wild type, thus conferring better survival under exposure to polymyxin B and colistin [50]. Also, EVs derived from *S*. *aureus* have been shown to be important for the survival of bacteria under ampicillin treatment [6]. Moreover, antibiotics themselves may stimulate EV production by various mechanisms depending on the nature of the antibiotic (reviewed in [24]).

Bacterial EVs may also contain quorum-sensing molecules that allow bacteria to communicate and coordinate group actions, such as quinolines or lactones [45,46].

Finally, EVs play a major role in bacterial survival against external threats. Because EV structure resembles that of bacteria, especially for gram-negative bacteria, EVs can act as decoys against bacteriophages, as observed with electron microscopy for *E*. *coli* and *Vibrio cholerae* [50,51].

## Bacterial EVs and interaction with human hosts

As with bacteria-to-bacteria communications, bacterial EVs mediate communications between bacteria and their hosts. The mechanisms of interactions between bacterial EVs and human host cells include EV interactions with host receptors, delivery of EV cargo into the host cell, and full incorporation of EVs into the host cell cytoplasm [8–10,29,58–61]. Molecular mechanisms of EV uptake still have to be understood. After bacterial EV adhesion/binding to host cells, 3 routes for the uptake of bacterial EVs into host cells have been proposed [12,44,58,59,62,63]: (i) Endocytosis, which is considered to be the main route of entry of bacterial EVs into eukaryotic cells; (ii) internalization of bacterial EVs through lipid rafts; and (iii) direct membrane fusion. However, the exact molecular mechanism of this latter route remains to be clarified.

Toll-like receptors (TLRs) were reported to be involved in the interactions of bacterial EVs with the host cells. It has been described that bacterial EVs from *M*. *catarrhalis* were internalized in human epithelial cells via interactions with TLR2 [64]. EVs derived from *Bifidobacterium* and *Lactobacillus* were found to enhance cellular TLR2/1 and TLR4 responses in dendritic cells [65]. The interaction of *Mycobacteria* derived EVs with mouse macrophages stimulated the release of cytokines and chemokines in a TLR2-dependent manner [66]. Also, bacterial EVs exposing LPS bind to host cells through interaction with cellular LPS-binding protein [12].

Whichever the exact mechanisms of EV-mediated interactions of bacteria with mammalian cells are, there is abundant evidence that many of the widely studied effects of bacteria on host organisms are indeed mediated by bacterial EVs (Fig 1D). One piece of such evidence is the ability of bacterial EVs to regulate immunomodulation and trigger related signaling cascades in host cells (Table 2) [57,62,67–75]. For instance, EVs generated in vitro by *S*. *aureus* up-regulate pro-inflammatory cytokines in vivo and facilitate a Th17 response [75]. EVs isolated from *Clostridium perfringens* up-regulate tumor necrosis factor (TNF), interleukin (IL)-6, and granulocyte colony-stimulating factor in in vitro experiments [57]. EVs derived from the gut bacterium *Akkermansia muciniphila* were shown to down-regulate the production of IL-6 from colon epithelial cells during colitis [68].

**Table 2:** Immunomodulatory effect of EVs.

Source of EVs	Effect of EVs
**Gram-negative**	
*Escherichia coli*	EVs containing shiga toxin (St2) induced caspase-9-mediated apoptosis and IL-8 secretion [67].
*Akkermansia muciniphila*	EVs ameliorated the production of a pro-inflammatory cytokine IL-6 from colon epithelial cells induced by *E*. *coli* EVs [68].
*Pseudomonas aeruginosa*	EVs-induced IL-8 secretion in primary human epithelial cells [69].
*Helicobacter pylori* *P*. *aeruginosa* *Neisseria gonorrhoeae*	EVs containing peptidoglycan up-regulated NF-κB and NOD1-dependent responses in vitro [62].
*Neisseria meningitidis*	In human neutrophils, EVs stimulated the secretion of TNF-α, IL-1β, IL-8, MIP-1α, and MIP-1β [70]. In human macrophages, EVs stimulated the production of IL-1β, TNF-α, IL-6, IL-12p40, IL-10, IL8, MIP-1α, MCP-1, and RANTES [71].
*Legionella pneumophila*	EVs showed to be potent pro-inflammatory stimulators of macrophages, acting via TLR2, IRAK-1, and NF-κB [72]. In lung alveolar epithelial cells, EVs up-regulated IL-6, IL-7, IL-8, IL-13, GM-CSF, IFN-γ, and MCP-1 [73].
*Brucella abortus*	In human monocytes, EVs modulated inhibited cytokine responses (TNF-α and IL-8) to *E*. *coli* LPS, Pam3Cys, or flagellin [74].
**Gram-positive**	
*Staphylococcus aureus*	EVs induced neutrophil recruitment and the production of MCP-1, RANTES, KC, MIP-2, and BAFF [75].
*Clostridium perfringens*	In RAW264.7 cells, EVs induced the secretion of inflammatory cytokines such as, G-CSF, TNF-α, and IL-6 [57].

EVs, extracellular vesicles; IL, interleukin; LPS, lipopolysaccharides; TNF, tumor necrosis factor.

Since EVs affect host immunity, EVs from pathogenic bacteria present in human microbiota may serve as a continuous natural vaccination of our organism. For example, EVs isolated from *Bacillus anthracis* and *Streptococcus pneumoniae*, as well as from *Mycobacterium tuberculosis*, have been shown to trigger an immune response. It is conceivable that this immune response may protect us from the development of disease. It also opens ways to use bacterial EVs as vaccines, as reported for *Neisseria meningitidis* [76,77].

The human body, and in particular human mucosae, are colonized by trillions of different bacterial strains [78]. EVs can help in the maintenance of epithelial integrity: EVs from certain commensal *E*. *coli* strains, specifically EcN and ECOR63, increase the host epithelial barrier function through up-regulation of ZO-1 and claudin-14 and down-regulation of the leaky protein claudin-2 [79].

Therefore, abnormal EV release may facilitate host pathologies either via direct interaction with host cells or indirectly by affecting the microbiota composition. Below, we discuss the role of bacterial EVs in different human mucosae.

### Oral microbiota–derived EVs

The oral microbiota, which is characterized by high microbial species diversity [80], represents one of the first lines of defense against pathogens. Although the role of EVs produced by symbiotic and commensal bacteria remains somewhat unclear, there have been several reports that EVs from an oral pathogen act as immunomodulators [81,82]. For instance, EVs derived from bacteria responsible for chronic periodontitis, *Porphyromonas gingivalis*, *Treponema denticola*, and *Tannerella forsythia*, were found to trigger pro-inflammatory host responses associated with periodontitis. These bacterial EVs induced NF-κB activation and up-regulated the secretion of TNFα, IL-8, and IL-1β [81]. Conversely, short RNAs present in EVs derived from *P*. *gingivalis*, *T*. *denticola*, and *Aggregatibacter actinomycetemcomitans* were found to down-regulate IL-5, IL-13, and IL-15 [82], suggesting that periodontopathogen-derived EVs exert multiple, often opposite, immunomodulatory functions. The immune-modulating properties of *Pseudomonas aeruginosa* EVs seem to be important not only in the oral cavity but also in the lungs, as it has been reported that in lung cells, *P*. *aeruginosa* release EVs carrying a specific sRNA capable of suppressing LPS-stimulated MAPK signaling, resulting in decrease of IL-8 levels in vitro and in vivo [69].

In contrast to the defensive role of bacterial EVs described above, they may also facilitate the infection of oral mucosa with other pathogens, such as HIV-1. Dong and colleagues have reported a novel mechanism of HIV-1 entry into nonpermissive cells, in which EVs of *P*. *gingivalis*, an invasive oral bacterium, interact specifically with HIV-1 and promote a CD4-independent HIV-1 entry into epithelial cells [83].

### Gut microbiota–derived EVs

It has been estimated that the human gut microbiota in healthy individuals contains in the range of 1,500 bacterial species, dominated primarily by Firmicutes and Bacteroidetes [84,85]. The normal gut microbiota plays an important role in host nutrient metabolism, maintenance of structural integrity of the gut mucosal barrier, immunomodulation, and protection against several pathogens [86]. It is conceivable that EVs derived from these bacteria can exert this protective effect on the host. In 2012, Shen and colleagues showed that the administration of EVs isolated from the commensal *Bacteroides fragilis* could mimic the immune tolerance to prevent inflammatory bowel disease that was produced by the administration of the bacteria itself [87]. Several other studies later confirmed that the key effects of the gut microbiota on host physiology and immunity are, in part, mediated by EVs released by these bacteria (reviewed in [5,88,89]). Indeed, EVs derived from gut microbiota, found in human blood and urine, have been shown to reflect the composition of this microbiota, demonstrating how bacterial EVs can be distributed throughout the human body either locally or systemically (reviewed in [10]).

Generally, EVs from probiotics have anti-inflammatory effects and promote immune tolerance. For example, EV-derived proteins from *Bifidobacterium longum* alleviate food allergy through mast cell suppression [88], while EVs from the probiotic *E*. *coli* Nissle trigger the production of IL-10 [90]. Similar anti-inflammatory properties, beneficial for the hosts, have been reported for EVs derived from different strains of gram-positive *Lactobacillus* [89,91–93].

In fact, bacterial EVs are now being considered as an alternative to probiotics in instances where the intestinal barrier is impaired or the use of live bacteria in the host could be dangerous, such as in immunocompromised individuals. EVs can penetrate through the mucus layer and interact with the host, without the risk of sepsis that live bacteria may present [94].

As far as the microbiota-related pathologies are concerned, bacterial EVs may also participate in the development of some of them. Recently, Zingl and colleagues showed that increased release of EVs upon infection allowed *V*. *cholerae* to rapidly modify their cell surface, thus evading host defenses and adapting to the gastrointestinal environment [95]. Moreover, patients diagnosed with intestinal barrier dysfunction, HIV-1, or cancer have a systemic increase of plasma bacterial EVs associated with LPS. The presence of these EVs, which correlate with impaired barrier integrity, is able to induce immune activation [17], a driving mechanism of HIV-1 disease [96]. Also, EVs derived from *Cutibacterium acnes*, *Propionibacterium acnes*, and *S*. *aureus* were implicated in the progression of skin inflammatory diseases such as atopic dermatitis and acne [97–100]. LPS in EVs from gram-negative and lipoteichoic acid in EVs from gram-positive bacteria can trigger inflammatory responses on host cells [11,101]. Bacterial EVs contain several toxins mimicking the physiopathology of their parental bacteria, such as shiga toxin from *E*. *coli* [67] and mycolactone toxin from *Mycobacterium ulcerans* [102].

### Vaginal microbiota–derived EVs

The healthy human vaginal microbiota is generally dominated by *Lactobacillus* spp. [103]. Lactobacilli are considered to be health-promoting bacteria that protect the vaginal environment from numerous uropathogens. For example, we previously reported that 2 *Lactobacillus* strains isolated from vaginas of heathy women, *Lactobacillus crispatus* and *Lactobacillus gasseri*, release EVs which protect human cells and tissues from HIV-1 infection [104]. We found that HIV-1 virions preincubated with *Lactobacillus*-derived EVs were less infectious for isolated cells and tissues than controls. Incubation of cells with these EVs did not affect infection. By using specific antibodies against functional gp120, we found that *Lactobacillus*-derived EVs make gp120 less accessible to HIV-1 target cells. Moreover, these *Lactobacillus*-derived EVs carry numerous bacterial metabolites and proteins that may be associated with the anti-HIV-1 effect [104].

EVs of *Gardnerella vaginalis*, which is the most virulent and predominant etiological agent for bacterial vaginosis, were demonstrated to be internalized by vaginal epithelial cells, resulting in cytotoxicity and in increase of pro-inflammatory IL-8 [105]. These effects were associated with the different virulence factors identified in *G*. *vaginalis*–derived EVs, in particular with vaginolysin, which had been demonstrated earlier to induce cytotoxicity, and with increase of IL-8 production in epithelial cells [106].

## Bacterial EVs in therapy

Because of their low cost, easiness to isolate and manipulate, and proven immunomodulatory properties, EVs derived from bacteria have been proposed as effective tools for vaccine development, drug delivery, and disease diagnoses [29,107]. Moreover, bacterial EVs can be concentrated in large amounts and stored for a long time.

One of the advantages of using bacterial EVs for vaccination is their ability to present multiple antigens simultaneously in a native state, thus inducing effective immune responses. As a result, EVs derived from many gram-negative bacteria (*N*. *meningitidis*, *V*. *cholerae*, and *Bordetella pertussis*) and gram-positive bacteria (*S*. *aureus*, *S*. *pneumoniae*, *Clostridium perfringes*, and *B*. *anthracis*) have been successfully used as vaccines or as adjuvants in vaccines [57,76,77,108–118]. The strategies to use bacterial EVs as vaccines include (i) direct administration of EVs purified from pathogenic bacterial cultures; (ii) EVs engineered to express single or multiple antigens on their surface; and (iii) extraction of the effector molecules from bacterial EVs and present them as proteolipidic vesicles (EV proteoliposomes) [76].

Successful use of EVs from gram-negative bacteria to generate antigen-specific humoral responses was described many years ago (reviewed in [76]). Recently, however, gram-negative–derived EVs have also been shown to induce antigen-specific CD8+ T-cell responses [119], which are essential in the case of therapeutic vaccination against tumors and intracellular viruses.

Moreover, immunization with EVs from gram-positive bacteria has also been reported to be successful: EVs from *S*. *aureus* were shown to induce specific antibodies and T-cell responses. Importantly, vaccination with *S*. *aureus*–derived EVs, in mice, conferred protection against lethality induced by airway challenge with a lethal dose of *S*. *aureus* and also against pneumonia induced by the administration of a sublethal dose of *S*. *aureus* [114]. Also, it has been reported that EVs derived from *S*. *aureus* coated with indocyanine green-labeled mesoporous silica nanoparticles against drug-resistant *S*. *aureus* infection provided an effective strategy against drug-resistant *S*. *aur*eus in infection [120].

In spite of all these promising data, several limitations need to be addressed before bacterial EVs can be safely used for vaccination: (i) Bacterial EVs may contain virulence/cytotoxic factors that might harm the recipient; (ii) it is difficult to ensure standardization of EV composition in each batch of EVs; and (iii) bacterial EVs have to be targeted to specific tissues [107]. In principle, EVs derived from gram-positive bacteria may be a better option in vaccine development, as they do not contain LPS and thus are generally less toxic than gram-negative bacteria-derived EVs [76].

Another possible application of EVs is drug delivery. Not only do EVs enhance drug uptake, but they also protect their cargo from degradation, thus delivering them in functional condition to target cells [121]. The loading of bioactive substances in bacterial EVs can be done in vivo or in vitro. In vivo, compounds of interest are encapsulated during EV biogenesis in cells treated with these compounds. In this case, during EV biogenesis, drugs inside the intracellular compartment can be encapsulated in the generated vesicles leading to a secretion of EVs loaded with the compound of interest. This approach was used for the creation of *P*. *aeruginosa*–derived EVs carrying gentamicin [122]. Alternatively, purified EVs could be loaded in vitro with different types of compounds through electroporation. This technique was successfully applied to load EVs with siRNA [123] or with gold nanoparticles [124].

Finally, bacterial EVs can be used for disease diagnosis. For example, EVs isolated from urine were used to sequence 16SrRNA in order to characterize the altered gut microbial populations in individuals with autism spectrum disorder [125]. A diagnostic usage of EVs has an important advantage because EVs travel, as shown, with microbiota from the gut and can be found in readily accessible body fluids. Also, they may provide the best insights to the links between microbiota and the health status of the host [10].

## Conclusions and perspectives: What we have to learn regarding bacterial EVs

Since their discovery, it is now clear that bacterial EVs are not useless cell debris but rather major players in important aspects of bacterial virulence, host immunomodulation, communication with other cells, survival, and other phenomena. However, despite the progress in our understanding of the role of bacterial EVs, the field requires tools and guidelines to better study these EVs (Table 3). One of the obstacles to progress in the field is a lack of standardized methodology in the isolation and purification of EVs, as well as a lack of well-defined identification of individual markers present on bacterial EVs [5]. EV composition and size can change drastically, depending on growth conditions [88,126,127] and on strains even within the same species [128].

**Table 3:** What we have to learn regarding bacterial EVs.

	What we know	What we do not know yet
**Bacterial EVs**	• Gram-positive and gram-negative bacteria release EVs. • Bacterial EVs are similar in size to mammalian EVs.	• Are bacterial EVs important for cell–cell communication? • What is the contribution of bacterial EVs in EV-derived human body fluids?
**Biogenesis/Secretion**	• The mechanisms of secretion of EVs derived from gram-positive and gram-negative are different.	• How is EV secretion regulated? • How do gram-positive EVs cross the peptidoglycan cell wall? • What are the factors influencing bacterial EV secretion? • How does the human immune system affect bacterial EV secretion? • Can we trace bacterial EVs to their cell of origin?
**Cargo** (Bioactive molecules)	• Bacterial EVs carry proteins, nucleic acids, lipids, metabolites, toxins, virulence factors, and LPS and LTA transmembrane proteins, which are found in gram-negative and gram-positive EVs, respectively.	• How is the cargo targeted to bacterial EVs? • Is the cargo specific to specific bacterial EVs?
**Functions**	• Play a role as immune modulators (TLR and NOD activation, cytokine secretion, and antigenic stimulation of immune cells) • Are important in bacterial communications (antibiotic resistance, quorum sensing, gene transfer, bacterial killing, and export of bacterial nutrients) • Play a role in bacterial pathogenesis (virulence factors, toxins, and bacterial decoys).	• Is bacterial EV secretion a mechanism of waste excretion? • Do all bacterial EVs have functions? • Do specific bacterial EVs target specific cells? • Does gram-positive EV uptake differ from gram-negative EV uptake?
**Interactions with other pathogens**	• *Lactobacillus-*derived EVs inhibit HIV-1 replication.	• Do bacterial EVs inhibit a wide range of pathogens?
**Diagnostic/Therapeutic**	• Diagnostic (EVs used for detection of active tuberculosis) • Vaccine (*Neisseria meningitidis* EVs used as vaccine against meningococci)	• Can bacterial EVs confer health benefit to the host? • Can bacterial EVs be used for targeted drug delivery? • Are pathogenic bacterial EVs safe as vaccine? • How can we ensure standardization of bacterial EV composition? • Can bacterial EVs be used as adjuvant in vaccination?
**Isolation**	• Bacterial EVs can be isolated using mammalian EV methods of isolation.	• Lack of consensus for production, isolation, and characterization • Lack of specific (surface) markers to identify bacterial EVs • What is the best method to isolate bacterial EV specifically adapted for gram-positive vs. gram-negative EVs?

EVs, extracellular vesicles; LPS, lipopolysaccharides; LTA, lipoteichoic acid; TLR, Toll-like receptor.

Also, we need reliable methods to trace EVs back to their mother cell of origin, very much like what is needed in the field of eukaryotic EVs. Tracing back EVs to their mother cells is important since in vivo EVs do not come from a homogenous cell population but rather are released by cells of many different types. Furthermore, the existing techniques still do not allow reliable and efficient separation of eukaryotic and prokaryotic vesicles present together in the same sample; this separation is needed to address bacterial EV heterogeneity. Recently, significant progress was made to overcome these limitations. In this regard, the methods to separate bacterial EVs from human body fluids by ultrafiltration, size-exclusion chromatography, and density gradient centrifugation have been recently described [17]. This may be one of the most promising approaches to discovering biomarkers and revealing pathophysiological mechanisms of particular diseases.

A better characterization of EVs will facilitate understanding of which types of EVs are predominantly secreted by certain bacteria and under which specific conditions. It may also increase our understanding of the possible functions of the different types of vesicles. The functions of these vesicles may not only be linked to their structure and biogenesis but also, and mostly, to the nature and extent of molecules that are exposed at their surface or present in their cargo.

Through the past decade, many studies have reported on the heterogeneity of the cargo. In 2021, we now have an overall idea of what bacterial EVs carry. These are membrane-bound molecules, cytoplasmic components such as nucleic acids, virulence factors, quorum-sensing molecules, immunomodulatory compounds, etc. The discovery of new components associated with EVs will help to reveal new functions of bacterial EVs. However, there are major limitations in these areas that need to be addressed in the future: One aspect of the cargo composition that is not fully understood yet is the mechanism of packaging these components in EVs. Is a given molecular component specific to some strains of bacteria, and is it specific to some EVs? Is it specific to certain environmental conditions? The ways and reasons why certain bacterial components are sorted into EVs remain to be elucidated.

Several studies have emphasized the importance of EVs and more specifically their cargo in the transfer of different bacterial components to other bacteria or host cells [46]. A very important problem that hasn’t been solved for eukaryotic EVs either is whether bacterial EVs are in fact targeting specific cells delivering a specific message to a particular cell.

In the context of quorum sensing, Toyofuku and colleagues not only showed that lactones travel from bacterium to bacterium through EVs but also that these EVs fuse with varying propensities to different bacteria, suggesting that the EVs are capable of recognizing particular cell types [46]. Whether this is an atypical example for bacterial EVs, or whether this is true for EVs carrying other cargos such as DNA, miRNA, or toxins, remains to be answered. Specific recognition patterns, such as ligand–receptor in the association of bacterial EVs and target cells, have not yet been identified [12]. Whether adhesins, which facilitate the interactions between bacterial EVs and target cells [129,130], can play the role of delivery to specific target cells remains somewhat unclear.

Recent data support the idea that bacterial EVs are more diverse than OMVs of gram-negative bacteria and membrane vesicles (MVs) of gram-positive bacteria. Different subtypes of bacterial vesicles have been reported [24–26]. What factors trigger specific OMV formation routes, versus factors triggering vesicle formation through blebbing or vesicle formation triggered through cell death (explosive cell lysis and bubbling cell death) [24]? Some factors such as antibiotics or nutrient limitation have been shown to favor one way over the other. Are there any other factors? Whether this is a general phenomenon for all bacteria also remains to be fully elucidated.

In summary, the science of bacterial EVs is as complex as that of eukaryotic EVs. Increasing our knowledge of bacterial EVs will not only add to our understanding of bacteria but also may potentially be used to expand the potential biotechnical use of bacterial EVs.
